# Current News

**Published:** 1894-10

**Authors:** 


### Current News.

### NEW ENGLAND DENTAL SOCIETY.

THE Thirty-second Annual Meeting of the New England Dental Society will be held in Hotel Clarendon, Tremont Street, Boston, Mass., Thursday and Friday, November 8 and 9, 1894, beginning Thursday afternoon at one o'clock. Please mark off the days on your appointment book *now*, and make an earnest effort to be present, as the meeting promises to be of unusual interest. A dinner is proposed with a light entertainment, Thursday, at 6.30 P.M. Programmes will be sent later.

Per order of the Executive Committee,

EDGAR O. KINSMAN, D.D.S.,
*Secretary.*

15 BRATTLE STREET, CAMBRIDGE, MASS.,
September 15, 1894.

### RECENT PATENTS.

A LIST of recent patents reported specially for the INTERNATIONAL DENTAL JOURNAL:

521,930.—Dental Plate. Thomas H. Graham, Toronto, Canada. Filed April, 6, 1894. Patented in Canada.

522,188.—Dental Articulator. George K. Bagby, Newberne, N. C. Filed April 23, 1894.

522,192.—Head-Rest. Arthur W. Browne, Prince's Bay, N. Y., assignor to the S. S. White Dental Manufacturing Company, Philadelphia, Pa. Filed February 13, 1893.

522,211.—Dental Abrading or Cutting Tool. Woodbury S. How, Philadelphia, Pa., assignor to the S. S. White Dental Manufacturing Company, same place. Filed August 16, 1893.

522,212.—Dental Bite-Plate. Woodbury S. How, Philadelphia, Pa., assignor to the S. S. White Dental Manufacturing Company, same place. Filed April 16, 1894.

522,291.—Angle Attachment for Dental Engines. Charles H. Davis, Worcester, Mass. Filed April 18, 1893.

522,309.—Dental Apparatus. William Wright, Boston, Mass., assignor of one-half to Granville O. Avery, same place. Filed April 17, 1894.

522,400.—Artificial Tooth. Edward Bowlus, Frederick, Md. Filed March 23, 1894.

522,552.—Adjustable Bracket for Dental Engines. Arthur W. Browne, Prince's Bay, New York, assignor to the S. S. White Dental Manufacturing Company, Philadelphia, Pa. Filed December 26, 1893.

522,563.—Head-Rest. William E. Hunt, Detroit, Mich. Filed June 15, 1892.

522,922.—Dental Chair. Dewell Stuck, Rochester, N. Y. Filed October 18, 1893.

Trade-Marks.—24,940.—Dental Gold-Foil. The S. S. White Dental Manufacturing Company, Philadelphia, Pa., New York and Brooklyn, N. Y., Boston, Mass., Chicago, Ill., and Atlanta, Ga. Filed November 13, 1893. Essential Feature the representation of a globe bearing a band on which are the words "Globe Gold-Foil."

24,941.—Dental Gum or Rubber. The S. S. White Dental Manufacturing Company, Philadelphia, Pa., New York and Brooklyn, N. Y., Boston, Mass., Chicago, Ill., and Atlanta, Ga. Filed November 13, 1893. Essential feature the words "Bow-Spring Rubber."

---

### UNION DENTAL CONVENTION.

The Twenty-sixth Union Dental Convention, of the Fifth, Sixth, Seventh, and Eighth District Dental Societies of the State of New York, will be held in the City of Buffalo, New York, October 30 and 31. A meeting of more than usual interest is expected.

D. H. Squire,
*Secretary.*

